# Detection of Low Blood Hemoglobin Levels on Pulmonary CT Angiography: A Feasibility Study Combining Dual-Energy CT and Machine Learning

**DOI:** 10.3390/tomography9040123

**Published:** 2023-08-18

**Authors:** Fernando U. Kay, Cynthia Lumby, Yuki Tanabe, Suhny Abbara, Prabhakar Rajiah

**Affiliations:** 1 Department of Radiology, University of Texas Southwestern Medical Center, Dallas, TX 75390, USA; suhny.abbara@utsouthwestern.edu; 2 Veterans Affairs North Texas Health Care System, Dallas, TX 75216, USA; cynthia.lumby@va.gov; 3 Department of Radiology, Ehime University, Matsuyama 790-0825, Japan; yuki.tanabe.0225@gmail.com; 4 Department of Radiology, Mayo Clinic, Rochester, MN 55901, USA; radpr73@gmail.com

**Keywords:** anemia, pulmonary embolism, computed tomography angiography, machine learning

## Abstract

Objectives: To evaluate if dual-energy CT (DECT) pulmonary angiography (CTPA) can detect anemia with the aid of machine learning. Methods: Inclusion of 100 patients (mean age ± SD, 51.3 ± 14.8 years; male-to-female ratio, 42/58) who underwent DECT CTPA and hemoglobin (Hb) analysis within 24 h, including 50 cases with Hb below and 50 controls with Hb ≥ 12 g/dL. Blood pool attenuation was assessed on virtual noncontrast (VNC) images at eight locations. A classification model using extreme gradient-boosted trees was developed on a training set (n = 76) for differentiating cases from controls. The best model was evaluated in a separate test set (n = 24). Results: Blood pool attenuation was significantly lower in cases than controls (*p*-values < 0.01), except in the right atrium (*p* = 0.06). The machine learning model had sensitivity, specificity, and accuracy of 83%, 92%, and 88%, respectively. Measurements at the descending aorta had the highest relative importance among all features; a threshold of 43 HU yielded sensitivity, specificity, and accuracy of 68%, 76%, and 72%, respectively. Conclusion: VNC imaging and machine learning shows good diagnostic performance for detecting anemia on DECT CTPA.

## 1. Introduction

Anemia is characterized by impaired delivery of oxygen to tissues due to a decrease in the number of red blood cells or in their oxygen-binding capacity [1]. It affects approximately one quarter of the world’s population [2], predisposing patients to negative health outcomes [3,4,5,6,7]. The prevalence of anemia is particularly high in emergency departments (ED) and intensive care units, ranging from 28% to 70% [8,9]. Anemia is associated with increased hospitalization, longer hospital stays, higher healthcare costs, higher disability rates, and decreased quality of life [10,11,12,13]. Although standard blood tests, such as complete blood count (CBC), are effective and quick in diagnosing anemia, there are scenarios where additional information obtained from imaging could provide more comprehensive insights into the patient’s condition. For example, in critically ill patients, having real-time data on hemoglobin levels through imaging could be vital. In addition, utilizing information from imaging for anemia screening can be resourceful, especially when the imaging is performed for other indications. Though medical imaging is not currently indicated to diagnose anemia, a few studies have shown the potential value of blood pool attenuation coefficients measured on noncontrast-enhanced computed tomography (CT) as a biomarker for detection of low blood hemoglobin (Hb) [10,14,15,16,17,18,19,20].

Pulmonary thromboembolism has contributed to the growing utilization of CT angiography (CTA) in ED [21]. Interestingly, anemia is known to be an independent risk factor for death in this population [22]. The possibility of detecting anemia on the same imaging modality could provide more holistic information for the management of such patients, particularly when CBC results are delayed or unavailable for critically ill patients. However, identifying anemia on pulmonary CTA has been historically challenged by the use of iodinated intravenous contrast. We hypothesized that virtual noncontrast (VNC) images derived from dual-energy CT (DECT) scanners [23] would allow for the identification of low Hb on pulmonary CTA. Additionally, with the advent of newer approaches, such as artificial intelligence and machine learning, which have recently emerged as tools for integrating information from multiple inputs into useful diagnostic outputs in radiology [24], we deemed them promising ancillary means to test our hypothesis.

Motivated by the perspective of combining new machine learning methods to leverage underutilized imaging data from pulmonary CTA, the aim of this study was to test if a classification model based on machine learning and multiple variables, including VNC blood pool CT attenuation, could detect subjects with low blood Hb levels among those who underwent DECT pulmonary CTA. As a secondary aim, we sought to determine if a simple measurement of the blood pool attenuation on VNC images could be used as a rapid screening tool for low blood Hb. If successful, this novel approach could serve as an adjunct to traditional blood testing, offering real-time and comprehensive data for patients, particularly in emergency and critical care settings.

## 2. Materials and Methods

### 2.1. Patient Selection

**Figure 1:**
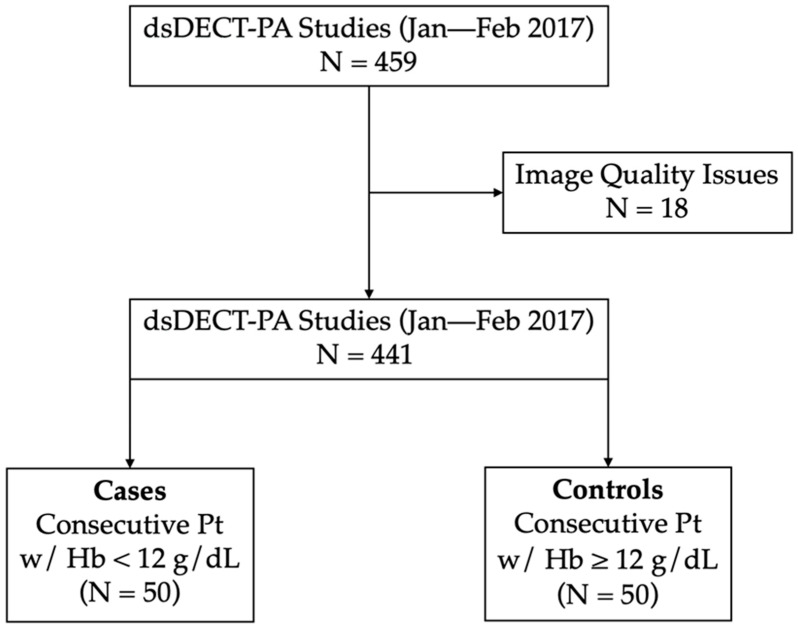
Study flowchart. Note: dsDECT-PA, dual-source dual-energy computed tomography angiography; Pt, patients; Hb, hemoglobin.

This is a single-institute retrospective feasibility study that was approved by the local institutional review board. Protected health information was handled on an as-needed basis following standard institutional ethical procedures on data confidentiality. The necessity for informed consent was waived. We searched the radiology report system for pulmonary CTA performed at our institution from 1 January through to 28 February 2017. Studies with poor or nondiagnostic quality as described in the radiological report (e.g., secondary to motion, excessive image noise, or suboptimal enhancement of the pulmonary arteries) were excluded. We consecutively reviewed the electronic medical records of the remaining cases and included 50 patients with blood Hb levels < 12 g/dL (cases) and 50 patients with values ≥ 12 g/dL (controls), as determined by complete blood counts collected within 24 h of the CTA, consecutively (Figure 1, study flowchart). Sample size was determined a priori for discovering an area under the receiver operating characteristics curve (AUC) of at least 0.65 with 80% power at a 5% significance level. The following patient variables were recorded from the CTA metadata: age, gender, and body mass index (BMI).

### 2.2. Dual-Energy CT Protocol

CTPA was obtained using second- or third-generation dual-source DECT technology (Somatom Definition Flash or Somatom Force, Siemens Healthineers, Erlangen, Germany). The two X-ray tubes were operated at 90–100 kVp (low-energy channel) and tin-filtrated 140–150 kVp (high-energy channel) with automatic tube current modulation. Scans started after intravenous injection of iohexol 350 mg-I/mL at 4–5 mL/s using a power injector (MEDRAD^®^ Stellant CT Injection System, Bayer, Whippany, NJ, USA), with a threshold of 120 Hounsfield units (HU) set at the main pulmonary artery bifurcation level. The CT scanner dose output (computed tomography dose index volume (CTDIvol)) and the volume of injected contrast medium were obtained from data reports stored on PACS.

### 2.3. Image Analysis

**Figure 2:**
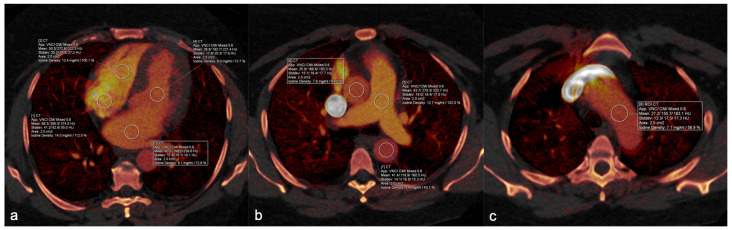
Anatomical sites of measurement. Illustrative figures of the anatomical regions where regions of interest were drawn to measure CT attenuation. Iodine mapping obtained by material decomposition analysis is displayed in colors, overlayed on the gray-scale image. Regions of interest: (**a**) right atrium [1], right ventricle [2], left atrium [3], left ventricle [4]. (**b**) Pulmonary artery [5], ascending aorta [6], descending aorta [7], and (**c**) aortic arch [8].

Two radiologists (C.L. and Y.T.) with 4 to 5 years of clinical experience and blinded to the Hb status of the patients independently measured the CT attenuation of blood pool on VNC and contrast medium (CM) using the *syngo*. (Siemens, Erlanger, Germany) via workstation (Siemens), which derives the CT attenuation of each component using a three-material decomposition algorithm [25]. Measurements were taken using circular regions of interest (ROI) with an approximate area of 2 cm^2^ at 8 different locations, carefully avoiding the vessel or cardiac walls and artifacts with the aid of simultaneous visualization of standard linearly blended reconstructions and iodine density maps. The sites of measurement were right atrium (RA), right ventricle (RV), pulmonary artery (PA), left atrium (LA), left ventricle (LV), ascending aorta (AscAo), aortic arch (AoArch), and descending aorta (DescAo), as demonstrated in Figure 2. The average of the measurements of the two readers was used for statistical and machine learning purposes.

### 2.4. Statistical Analyses and Machine Learning Modeling

All of the analytical steps and machine learning modeling were performed on R (version 3.5.3, the R Foundation for Statistical Computing, Vienna, Austria) using native built-in functions and the packages “caret” [26] and “pROC” [27]. Normality of data was tested using the Shapiro–Wilk test. The differences in patient demographics, scanner output, and CT attenuation numbers between cases and controls were tested using unpaired Student’s t- and chi-squared tests for normally distributed continuous and categorical variables, respectively. Inter-reader agreement between the CT measurements was tested using Bland–Altman analysis [28]. The significance level for α-type errors was set to 5%.

An extreme gradient-boosted trees algorithm with dropout regularization (xgbDART) [29] was selected as the foundation for building a classification model to detect low blood Hb levels based on the collected variables (demographical: gender, age, and BMI; study parameters: CTDIvol and volume of iodinated contrast injected; CT attenuation obtained at eight different regions: VNC and CM components). Data elements were deidentified upon dataset entry. There were no missing data elements; no data preprocessing steps were taken. The full dataset of 100 cases and controls was randomly split into training/validation (n = 76) and test (n = 24) subsets using stratification by low blood Hb level status. Laboratorial quantification of the Hb blood sample was used as ground truth. The algorithm was iteratively trained using 10-fold cross-validation in the 76 training/validation instances, with performance metric set to maximization of the AUC for discriminating patients with Hb < 12 g/dL from those with Hb ≥ 12 g/dL. Hyperparameters were fine-tuned using random search over 200 iterations. The hyperparameters that maximized the cross-validation AUC were 930 boosting iterations, maximum tree depth was 5, shrinkage factor was 0.04, minimum loss reduction was 8.13, subsample percentage was 0.58, subsample ratio of columns was 0.41, fraction of trees dropped was 0.36, probability of skipping dropout was 0.30, and minimum sum of instance weight was 0. The best model developed in the training/validation step was used to generate class probabilities for low Hb on the test set, which were subsequently evaluated using receiver operating characteristics (ROC) analysis. We estimated 95% confidence intervals (CI) for the area under the ROC curve using the De Long method. Bootstrapping with 2000 iterations was used to determine median and 95% CI estimates for sensitivity, specificity, accuracy, false positive, and false negative intervals at the point closest to the top left of the ROC graph.

To ascertain the practicality of introducing a more streamlined screening instrument for the identification of reduced blood Hb levels, we planned to make a post hoc selection of the most influential covariate in the machine learning model. This determination was based on the ranking derived from the relative feature importance as presented by the xgbDART model. Once identified, this predominant covariate underwent a process of univariate linear regression analysis, followed by ROC assessment, applied across the complete sample of 100 patients.

## 3. Results

### 3.1. Characteristics of the Sample

A total of 100 adult patients who presented at the ED with symptoms and signs of pulmonary embolism and who were referred for pulmonary CTA were included in the study. The mean age of the patients was 51.3 years with a standard deviation of 14.8 years. The ratio of male to female patients was 42:58. The summary of patient variables, CT dose output, and contrast volume is displayed in Table 1. No significant differences were noted between cases and controls. Table 2 summarizes the patient variables according to the split between the training and test subsets for machine learning purposes. No significant differences were noted between the two subsets.

### 3.2. Image Analysis

The mean measurement bias on VNC-CT attenuation between the two radiologists ranged from −2.21 (AscAo) to 0.48 (LV) HU (Table 3). Among cases, mean VNC-CT attenuation ranged from 39.09 (DescAo) to 50.04 (PA) HU, while among controls, values ranged from 45.35 (AoArch) to 57.63 (PA) HU (Table 4). VNC-CT attenuation measures at all locations, excepting the RA (*p* = 0.06), were significantly lower in cases when compared to controls (*p* < 0.01).

### 3.3. Machine Learning Modeling

**Figure 3:**
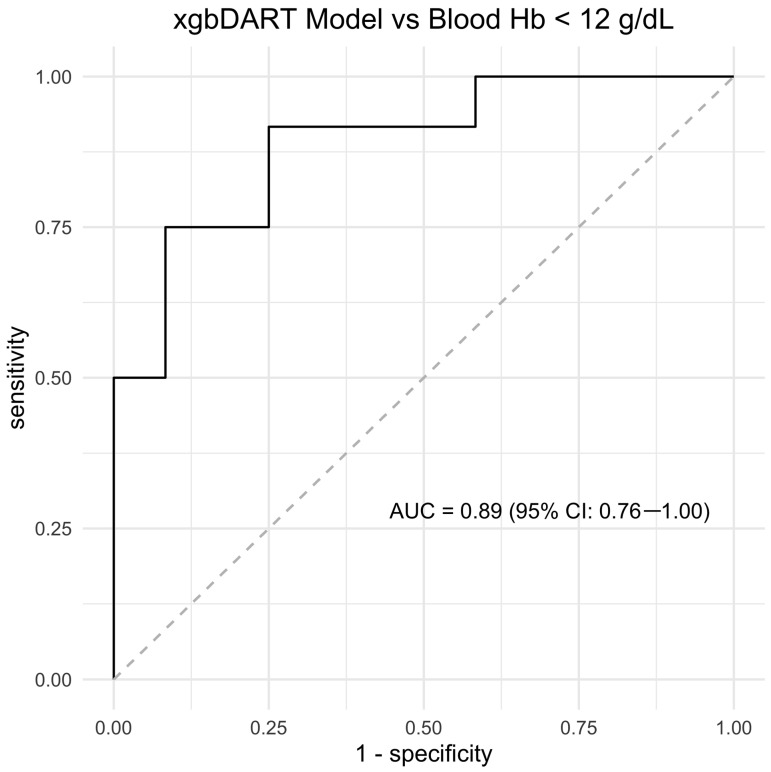
Receiver operator curve analysis of the output probabilities from the machine learning model for detecting blood hemoglobin (Hb) less than 12 g/dL. Note: xgbDART, extreme gradient-boosted trees with dropout regularization model; AUC, area under the curve; CI, confidence interval.

**Figure 4:**
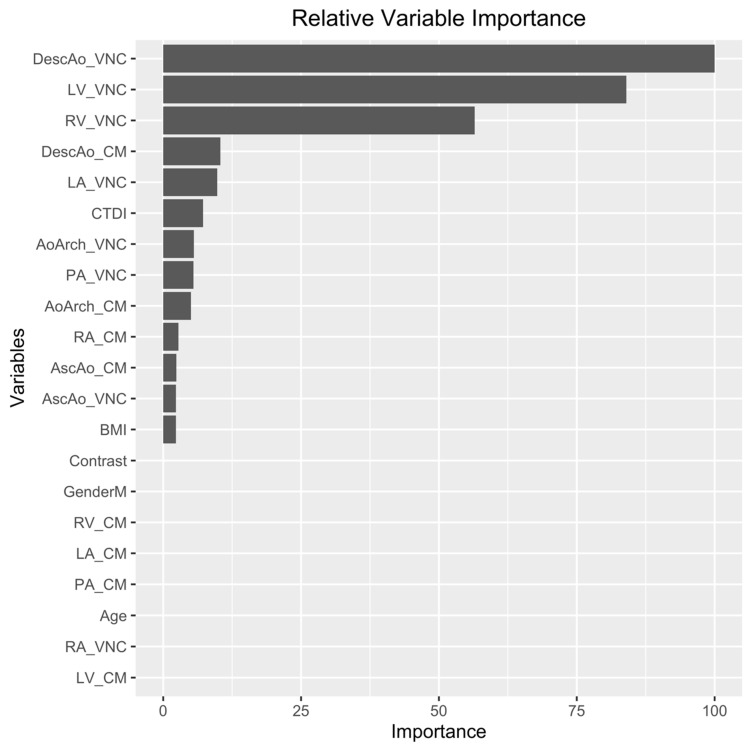
Bar chart showing the relative variable importance in the machine learning model. Note: RA, right atrium; RV, right ventricle; LA, left atrium; LV, left ventricle; Asc Ao, ascending aorta; Desc Ao, descending aorta; PA, pulmonary artery; Ao Arch, aortic arch; VNC, virtual noncontrast CT attenuation component; CM, contrast medium CT attenuation component.

The AUC of the best performing model during training was 0.83 (95% CI: 0.74–0.92). The sensitivity, specificity, and accuracy of the xgbDART model for detecting low blood Hb in the test set was 0.83 (95% CI: 0.67–1.00), 0.92 (95% CI: 0.67–1.00), and 0.88 (0.71, 0.96), respectively, with a median number of 1 false positive (95% CI: 0–4) and 2 false negatives (95% CI: 0–4) within the 24 test cases (Figure 3, ROC curve). DescAo VNC-CT attenuation had the highest relative importance among all model features (Figure 4, bar figure chart).

### 3.4. Univariate Analysis of the Top-Ranked Variable

**Figure 5:**
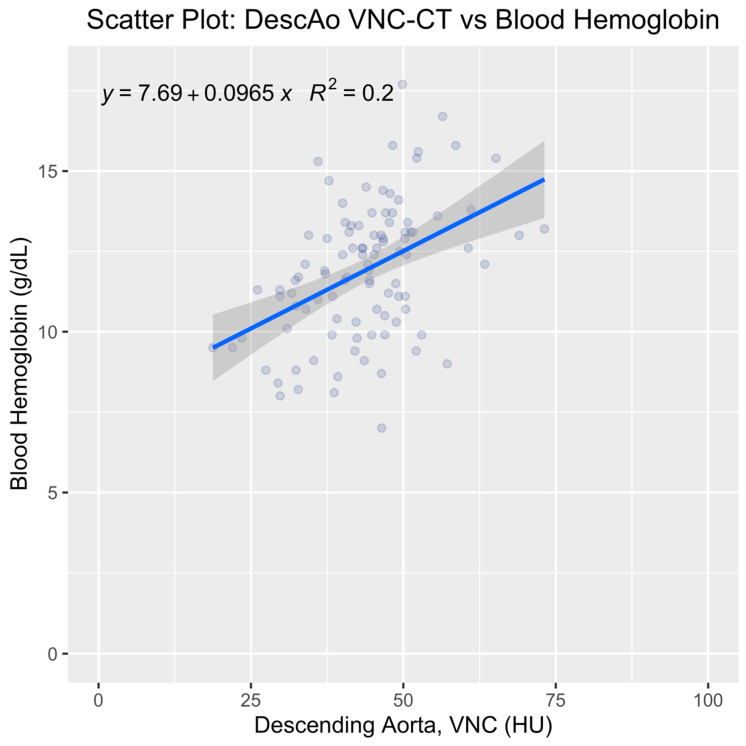
Scatter plot of left ventricular virtual noncontrast CT attenuation (LV VNC-CT) versus blood hemoglobin (Hb). Trendline obtained by linear regression in blue, with 95% confidence intervals shown as grey bands.

**Figure 6:**
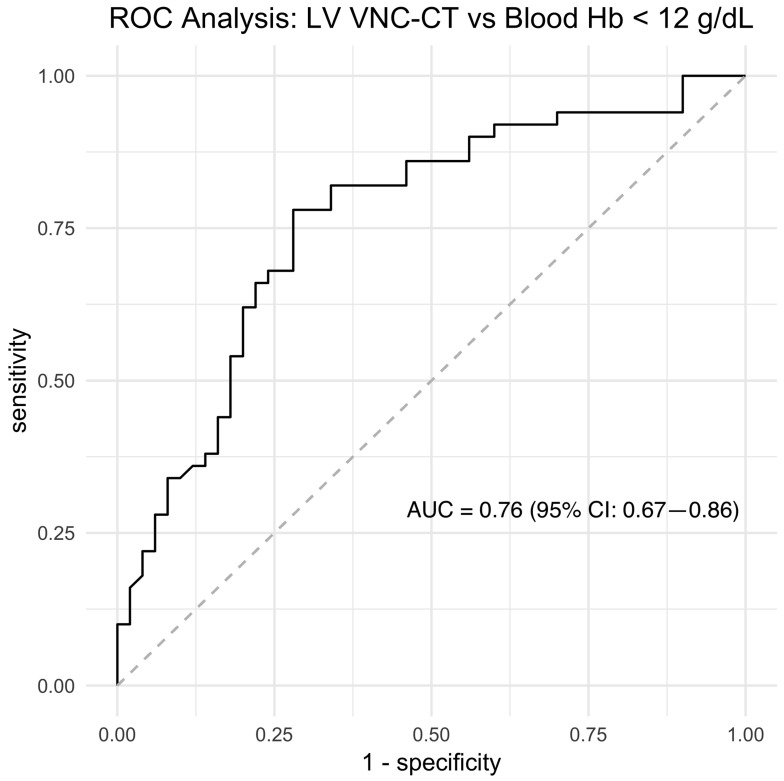
Receiver operator curve analysis of descending aorta virtual noncontrast CT attenuation number for diagnosing blood hemoglobin (Hb) less than 12 g/dL. Note: AUC, area under the curve; CI, confidence interval.

For the univariate analysis, we aimed to evaluate the linear relationship between blood Hb and DescAo VNC-CT attenuation, which was identified as the top-ranking variable by the machine learning model. It is important to note that this analysis was performed on the entire patient cohort to maximize statistical power and robustness, and it was conducted independently of the machine learning model. In this analysis, blood Hb was the dependent variable (y) and DescAo VNC-CT attenuation was the independent variable (x), resulting in the equation y = 0.097x + 7.69, with R^2^ = 0.20 (Figure 5, scatter plot). The AUC for detecting low blood Hb using DescAo VNC-CT attenuation as a singular variable was 0.76 (95% CI: 0.67–0.86) (Figure 6, ROC curve). The optimal DescAo VNC-CT attenuation threshold was determined to be 42.82 HU (95% CI: 39.65–46.58), yielding a sensitivity of 0.68 (95% CI: 0.52–0.84), specificity of 0.76 (95% CI: 0.58–0.84), and accuracy of 0.72 (95% CI: 0.64–0.80), with a median number of false positives of 12 (95% CI: 4–21) and false negatives of 16 (95% CI: 8–24) among the 100 patients. This univariate analysis allowed us to understand the raw association between blood pool attenuation in DescAo and blood Hb levels without the influence of other variables considered in the machine learning model.

## 4. Discussion

This study has demonstrated the feasibility of a novel approach based on machine learning for detecting low Hb levels on CTPA performed in a DECT scanner. The results of this paper add to the repertoire of opportunistic screening capabilities of CT scans. Opportunistic screening is an important emerging concept in radiology that adds value to imaging by optimizing extraction of convenient data. These data can be extracted by semiautomated or manual techniques to provide quantitative information on several diseases, which can be used for screening purposes. There has been an increase in utilization of this concept in recent years due to the maturation of artificial intelligence technologies [30], which provides automatic data extraction without additional time, cost, or radiation. Using deep learning algorithms, body composition parameters such as aortic calcification, muscle density, ratio of visceral to subcutaneous fat, liver fat, and bone mineral density can be extracted from routine CT scans. These parameters outperform established clinical parameters to predict future cardiovascular events, such as stroke, myocardial infarction, and death [31]. Such underutilized data could allow early disease detection and risk stratification, resulting in early treatment.

Anemia is often an unrecognized and undertreated entity with higher prevalence in several populations. The diagnosis of anemia has several clinical implications, including increased morbidity, mortality, hospitalization, length of hospitalization, and healthcare costs as well as decreased efficacy of chemotherapy/radiotherapy [32]. Specifically, in patients with acute PE, presence of anemia has been shown to be an independent predictor of mortality, with a hazard ratio of 1.16 for each 1 g/dL decrease in Hb. Patients with anemia also have a higher risk of fatal PE and worse survival [22]. Besides anemia, another potential use of DECT is to retrospectively provide image-based estimates of blood hematocrit values, which are necessary for the calculation of myocardial tissue parameters on delayed contrast-enhanced cardiac CT [33].

Studies that evaluated the capability of CT for detecting anemia were carried out on noncontrast scans. The attenuation of blood in a noncontrast CT is linearly dependent on Hb concentration, approximately 1.85 HU per gram of Hb per 100 mL of blood [34]. Hence, a low attenuation in noncontrast CT is an indicator of anemia. Early studies used the visibility of interventricular septum (IVS) relative to a low attenuation blood pool as a reliable indicator of severe anemia [14,18]. This sign was positive in all cases with Hb in the range of 7.6 to 10.2 gm/dL [16]. Hyperattenuation of the aortic wall (aortic ring sign) was found to be more sensitive in detecting anemia [20]. For quantitative purposes, a threshold of ≤35 HU has a large AUC (0.89), sensitivity (84%), and specificity (94%) in the detection of anemia [20]. A combination of subjective and objective analyses provides the best trade-off between sensitivity and specificity [20]. Differences in CT attenuation between IVS and LV can also detect severe anemia. One study used a cut-off of >13.5 HU [10], whereas other studies used >6–8 [15] and >10–12 [35] HU for the diagnosis of severe anemia. Attenuation of dural venous sinus confluence also has a direct positive correlation with Hb, with attenuation of <42.35 able to detect anemia <10 mg/dL [36]. Jung et al. evaluated anemia on CTPA using the attenuation values from a single noncontrast slice used for contrast bolus tracking purposes. A cut-off of 50 HU in the ascending aorta for men and 43 HU in the descending aorta for women was used for diagnosing anemia with sensitivity of 80% for men and 91% for women and specificity of 84% for men and 85% for women [37]. Noticeably, the diagnostic performance of our machine learning algorithm (i.e., sensitivity and specificity of 83% and 92%) closely matched that found by Jung et al. [37] using a different approach, as did the best blood pool CT attenuation threshold measured at the descending aorta on VNC (i.e., 43 HU). Notwithstanding, the goodness of fit of the linear model for our univariate linear regression mode is only modest in comparison to that found by Jung et al. [37] (R^2^ of 0.20 versus 0.54, respectively). This discrepancy could be explained by inherent differences on how VNC images are generated in comparison with true noncontrast images (TNC).

In DECT, VNC images are generated from contrast-enhanced CT by a process of material decomposition of iodine and water, in which the iodine content of pixels is removed. The VNC images are analogous to TNC and can be used to obviate the necessity of TNC in multiphasic CT studies [38,39]. The attenuation values of VNC are approximately those of TNC but are not the same. The differences between the VNC and TNC are <15 HU in 91.5% to 92.6% [40,41], with mean difference of −3.6 + 8.3 HU [42]. There is incomplete removal of iodine and higher attenuation in VNC images that are derived from arterial-phase postcontrast images when compared to the venous-phase ones due to dense concentration of iodine [41]. Incomplete elimination of contrast in the thoracic aorta in dual-source CT near the heart can also be affected by pulsation of the aorta and pulmonary arteries, which could result in mild spatial misalignment. Due to this reason, the threshold or cut-offs that are used in TNC cannot be automatically translated for use in VNC. In our study, we provide optimal cut-off and location for diagnosing anemia on DECT CTPA.

Our study emphasizes the growing potential of advancements in DECT technology, particularly when integrated with the power of machine learning algorithms. The aim is to provide healthcare professionals with a robust platform for opportunistic screening, ensuring that clinical conditions, which might previously have been overlooked or undetected in early stages, are now recognized promptly. As multienergy CT technology continues to advance, there are emerging tools, such as photon-counting CT, that have also demonstrated capabilities to estimate blood Hb levels [43]. As we move into an era of integrated healthcare, it is foreseeable that the future will see sophisticated informatic tools becoming an intrinsic part of electronic medical record systems. By drawing information from various data sources, including these opportunistic screening tools, it becomes possible to provide a comprehensive overview of a patient’s health status. This ensures that conditions such as anemia are flagged early on, allowing patients to receive the necessary medical attention without delay.

Our study has a few limitations. This is a proof-of-concept study in a small number of patients from a single center. It will need validation with larger, multicenter studies. It also needs validation in other DECT scanners and across different cut-off values for diagnosing anemia. Our sample of subjects were scanned in second- and third-generation dual-source scanners, which limit the generalizability of our measurements to different DECT technologies. In addition, while we have used a single threshold to diagnose anemia, the World Health Organization establishes different Hb cut-off levels for diagnosing anemia in men and women (13 and 12 g/dL, respectively). A larger training set may be also required to improve the accuracy of the method and ensure applicability to a wider population. Machine learning algorithms have the capabilities to extract attenuation values in the aorta and the heart, which will be evaluated in future studies. Lastly, the clinical impact of recognizing anemia on CTPA needs to be evaluated in large outcome studies. Notwithstanding, the results of this study shed some light on the capability of DECT in providing information about low Hb levels, even in contrast-enhanced studies.

In conclusion, there is currently underutilized data on DECT pulmonary CTAs performed in patients with suspected pulmonary embolism in the ED, which could be used to screen for anemia either using advanced machine learning modeling or more simply by measuring the CT attenuation values of the blood pool in the descending aorta on VNC images.

## Figures and Tables

**Table 1:** Demographic and CT acquisition variables: cases versus controls.

Variables	Cases	Controls	*p*-Value
Age (years)	50.3 ± 15.4	52.3 ± 14.3	0.51
Male-to-female ratio	22/28	20/30	0.84
BMI (kg/m^2^)	29.9 ± 9.9	30.1 ± 9.5	0.88
RBC (million cells/mcL)	3.6 ± 0.5	4.6 ± 0.5	<0.001
Hematocrit %	31.3 ± 3.7	40.6 ± 3.2	<0.001
Hb (g/dL)	10.2 ± 1.2	13.6 ± 1.3	<0.001
CTDI (mGy)	8.1 ± 4.5	9.6 ± 4.8	0.12
Contrast volume	85.9 ± 22.1	84.1 ± 13.8	0.63

Note: BMI, body mass index; RBC, red blood cells; Hb, hemoglobin; CTDI, computed tomography dose index.

**Table 2:** Demographic and CT acquisition variables: Training versus test partitions for ML.

Variables	Training	Test	*p*-Value
Age (years)	50.1 ± 13.9	55.2 ± 16.9	0.13
Male-to-female ratio	33/43	9/15	0.64
BMI (kg/m^2^)	29.4 ± 9.5	31.8 ± 10.0	0.29
RBC (million cells/mcL)	4.10 ± 0.7	4.16 ± 0.7	0.72
Hematocrit %	35.9 ± 5.7	36.1 ± 6.3	0.92
Hb (g/dL)	11.9 ± 2.1	11.9 ± 2.2	0.98
CTDI (mGy)	8.5 ± 4.2	10.0 ± 5.8	0.24
Contrast volume	84.3 ± 20.1	87.2 ± 11.4	0.37

Note: ML, machine learning; BMI, body mass index; RBC, red blood cells; Hb, hemoglobin; CTDI, computed tomography dose index.

**Table 3:** Average bias and limits of agreement of CT attenuation measurements between the two readers.

ROI Location	Average Bias	97.5th Limit	2.5th Limit
RA	−0.64	36.86	−38.13
RV	−0.17	23.84	−24.18
LA	−0.45	18.50	−19.40
LV	0.48	17.51	−16.56
AscAo	−2.21	17.56	−21.98
DescAo	−0.68	10.96	−12.32
PA	−0.43	17.39	−18.24
AoArch	0.23	16.22	−15.77

Note: ROI, region of interest; RA, right atrium; RV, right ventricle; LA, left atrium; LV, left ventricle; AscAo, ascending aorta; DescAo, descending aorta; PA, pulmonary artery; AoArch, aortic arch.

**Table 4:** Average CT attenuation values at different levels of the cardiovascular system: cases versus controls.

Location	Component	Cases (HU)	Controls (HU)	*p*-Value
RA	VNC	48.14 ± 12.97	53.01 ± 12.73	0.06
	CM	133.49 ± 271.17	247.73 ± 97.44	0.14
RV	VNC	46.97 ± 11.85	56.12 ± 12.08	<0.001
	CM	250.34 ± 105.32	245.54 ± 85.54	0.80
LA	VNC	43.02 ± 9.84	51.62 ± 10.30	<0.001
	CM	180.67 ± 50.21	182.65 ± 60.75	0.86
LV	VNC	42.75 ± 8.91	52.42 ± 11.33	<0.001
	CM	165.61 ± 50.54	163.37 ± 55.27	0.83
Asc Ao	VNC	41.09 ± 11.52	46.89 ± 9.63	<0.01
	CM	169.61 ± 54.83	158.25 ± 63.74	0.34
Desc Ao	VNC	39.09 ± 8.75	48.24 ± 8.44	<0.001
	CM	154.05 ± 64.81	133.32 ± 61.66	0.10
PA	VNC	50.04 ± 14.13	57.63 ± 13.48	<0.01
	CM	239.87± 96.01	238.06 ± 84.39	0.92
Ao Arch	VNC	40.94 ± 9.26	45.35 ± 7.18	<0.01
	CM	162.19 ± 63.91	147.36 ± 69.73	0.27

Note: Third and fourth columns, mean ± standard deviation; RA, right atrium; RV, right ventricle; LA, left atrium; LV, left ventricle; Asc Ao, ascending aorta; Desc Ao, descending aorta; PA, pulmonary artery; Ao Arch, aortic arch; VNC, virtual noncontrast component; CM, contrast medium component.

## Data Availability

Patient data cannot be shared under the approved study protocol.
